# The Influence of Residual Alveolar Bone Height on Graft Composition after Maxillary Sinus Augmentation Using Two Different Xenografts: A Histomorphometric Comparative Study

**DOI:** 10.3390/ma13225093

**Published:** 2020-11-11

**Authors:** Silvio Taschieri, Moses Ofer, Stefano Corbella, Tiziano Testori, Claudia Dellavia, Carlos Nemcovsky, Elena Canciani, Luca Francetti, Massimo Del Fabbro, Gianluca Tartaglia

**Affiliations:** 1 Department of Biomedical, Surgical and Dental Sciences, Università degli Studi di Milano, 20123 Milan, Italy; silvio.taschieri@unimi.it (S.T.); stefano.corbella@unimi.it (S.C.); info@tiziano-testori.it (T.T.); claudia.dellavia@unimi.it (C.D.); elena.canciani@unimi.it (E.C.); luca.francetti@unimi.it (L.F.); gianluca.tartaglia@unimi.it (G.T.); 2 Dental Clinic, IRCCS Istituto Ortopedico Galeazzi, 20161 Milano, Italy; 3 Department of Oral Surgery, Institute of Dentistry, I. M. Sechenov First Moscow State Medical University, 119146 Moscow, Russia; 4 Department of Periodontology & Dental Implantology, School of Dental Medicine, University of Tel Aviv, 6997801 Tel Aviv-Yafo, Israel; mosesofer@gmail.com (M.O.); carlos@tauex.tau.ac.il (C.N.); 5 Department of Periodontics and Oral Medicine, The University of Michigan, School of Dentistry, Ann Arbor, MI 48109, USA

**Keywords:** histomorphometric analysis, maxillary sinus augmentation, new bone formation, residual bone height, xenograft

## Abstract

Aim: To evaluate the hypothesis of a correlation between the preoperative residual alveolar bone height (RBH) and graft maturation after maxillary sinus floor augmentation procedures using two different bone substitutes. Methods: A total of 20 patients who underwent unilateral maxillary sinus floor augmentation with either mineralized deproteinized bovine bone (DBBM) or a xenograft enriched with polymer and gelatin (NBS) were included in this prospective study. Six months after sinus surgery, bone biopsies were harvested with a 3.2 mm diameter trephine bur, prior to dental implant placement. Histomorphometric analysis was performed, and the results were correlated with the individual RBH. Implants were loaded after 5 months of insertion, and 1-year implant success and marginal bone level change were assessed. Results: RBH was 2.17 ± 1.11 mm (range 0.5–3.5 mm) and 2.14 ± 0.72 mm (range 0.5–3.0 mm) in the NBS and DBBM group, respectively. The biopsy analyses for the DBBM group showed woven bone increases by 5.08% per 1-mm increment of RBH; medullary spaces decreased by 9.02%, osteoid decreased by 4.4%, residual biomaterial decreased by 0.34%, and lamellar bone increased by 5.68% per 1-mm increase of RBH. In the NBS group, samples showed woven bone increases by 8.08% per 1-mm increase of RBH; medullary spaces decreased by 0.38%; osteoid increased by 1.34%, residual biomaterial decreased by 0.58%, and lamellar bone decreased by 5.50% per 1-mm increase of RBH. There was no statistically significant difference in the correlation between RBH and lamellar bone, woven bone, and osteoid, independently of the material used. Implant success was 100% in both groups, and marginal bone loss was 1.02 ± 0.42 mm in DBBM and 0.95 ± 0.31 mm in the NBS group after the 1-year follow-up. Conclusion: In spite of the absence of significance, the observed trend for woven bone to increase and medullary spaces to decrease when RBH increases deserves attention. Residual bone dimension might be a determinant in the bone graft maturation after maxillary sinus augmentation.

## 1. Introduction

Oral rehabilitation with osseointegrated implants is a successful and widely used treatment option for patients presenting partial or edentulous arches. Following tooth extraction, bone resorption process (also in some areas leading to pneumatization of the maxillary sinuses) could result in limited bone volume, thus requiring bone augmentation procedures allowing placement of dental implants [1,2]. In cases of inadequate bone volume in the posterior maxilla, the treatment protocols will involve maxillary sinus floor augmentation using various techniques and biomaterials. [3,4]. Among them, autogenous bone is considered by some authors as a “gold standard” as the most effective, nonimmunogenic, osteoinductive, and osteoconductive biomaterial and a source of osteoprogenitor cells and growth factors [5,6].

The possibility of using numerous grafting materials other than autogenous bone, such as allografts, alloplasts, xenografts, bone substitutes, and blood clots alone or in combinations or hybrid enriched materials, offers a great variability to overcome the major limitation of the use of autogenous bone—it often requires two-site surgery, which increases the patient postoperative morbidity [7,8].

The survival rates of implants placed in augmented sinuses are similar to those placed in the native bone in the posterior maxilla and remain relatively high [9,10,11,12]. The sinus-lift technique, selection of grafting materials, and the timing of implant placement have an impact on the bone remodeling [13,14,15]. The amount of newly formed bone is crucial for the evaluation of grafting materials by measuring the apposition percentage of newly formed bone. Successful osseointegration of the placed implant in the augmented area is dependent on functional remodeling and replacement of the grafting material by vital bone tissue [16,17]. A higher new bone volume and bone density results in a higher contact of the implant with the bone and contributes to higher implant stability and survival rates [17].

Selection of the ideal grafting material for the sinus augmentation is a debatable matter. However, histomorphometric analyses of maxillary sinus lift show that the amounts of newly formed bone, residual graft particles, and soft tissue components (bone marrow and/or connective tissue) differ with the use of different grafting materials [14,16,18]. In addition to the above-mentioned factors, untreated systemic disease, smoking status, or anatomical variances play an important role in the regenerative results [15].

Delayed or insufficient bone maturation after the sinus lift, and therefore delayed bone remodeling and replacement of grafting material by bone, can also be caused by the size of the sinus cavity and can occur in sinuses of larger dimensions or in cases of limited residual alveolar bone after tooth loss [19]. A strong negative correlation between buccopalatal thickness and the percentage of newly formed vital bone has been described [19], indicating that more time may be needed to allow proper vital bone formation in large sinus cavities.

On the other hand, very limited literature focuses on a possible relationship between histomorphometric fractions and height of residual bone before the sinus lift procedure, which is rarely mentioned as a secondary observation. This may be partially caused by the fact that no such correlation is observed, or it is not analyzed and therefore reported. We hypothesized that the preoperative bone height of the subantral residual alveolar bone is correlated with bone maturation and graft replacement after the sinus augmentation. Consequently, each postoperative histomorphometric compartment (lamellar bone, woven bone, osteoid, biomaterial particles, and medullary spaces) might be predicted based on the preoperative height of residual subantral alveolar bone. The aim of the present study was to investigate the hypothesis of a correlation between the preoperative residual alveolar bone height and the maturation of graft through the histomorphometric analysis of bone biopsies taken from patients who underwent sinus floor augmentation using two different xenografts.

## 2. Materials and Methods

The study protocol was approved by the Scientific Board of the IRCCS Istituto Ortopedico Galeazzi in Milan, Italy. All patients signed an informed consent form before the procedure and agreed to be part of the study performed in accordance with the Good Clinical Practice and the Declaration of Helsinki. Inclusion criteria were as follows:

1. ASA-1 or ASA-2, following the classification proposed by the American Association of Anaesthesiologists;
2. Single or multiple edentulism in lateroposterior area of the maxilla (premolars/molars);
3. Less than 5 mm of residual alveolar bone height (RBH);
4. No previous regenerative procedures in the site of intervention;
5. Nonsmokers, former smokers, or smoking less than five cigarettes a day;
6. No sinus pathology.

In total, 20 patients were included in this prospective study. Each patient underwent a single maxillary sinus floor augmentation procedure, with the lateral approach. In total, 10 sinuses were grafted using mineralized deproteinized bovine bone (DBBM, Bio-Oss, Geistlich Pharma AG, Wolhusen, Switzerland), while the other 10 were grafted with a xenograft enriched by polymer and gelatin (NBS, SmartBone, Industrie Biomediche Insubri S/A, Mezzovico-Vira, Switzerland) by a single experienced surgeon.

The patients were allocated alternatively to DBMM or NBS group.

### 2.1. Surgical Protocol

The same sample was used, and the same surgical protocol and histomorphometric analysis were performed, as in a recent publication by Taschieri et al. [20]. In brief, each patient underwent preoperative cone beam computed tomography (CBCT) scanning. The height of the residual alveolar bone was assessed by a single operator. The sinus floor elevation was performed via a full-thickness trapezoidal flap. After the Schneiderian membrane was detached and elevated coronally, the cavity underneath was filled with either NBS or DBMM followed by the lateral window coverage using a collagen membrane (Alpha-Bio’s GRAFT collagen membrane, Alpha-Bio Tec, Kiryat Arye, Petach Tikva, Israel), and then the flap was sutured with interrupted sutures (Ethilon, Ethicon, Inc., Johnson & Johnson, Piscataway, NJ, USA).

Postoperative care consisted of 0.2% chlorhexidine digluconate solution twice a day for 10 days, nonsteroidal analgesics as needed, and antibiotic therapy (amoxicillin 1 g twice a day for 6 days).

The radiographic and clinical evaluation and implant placement were performed 6 months after the sinus augmentation. The bone samples were retrieved with a 3.2 mm diameter trephine bur followed by a dental implant placement (Alpha-Bio Tec, Kiryat Arye, Petach Tikva, Israel). The obtained bone sample was fixed in 40% ethanol solution for 48 h at 4 °C and then dehydrated with ethanol, propanol, and xylene for 48 h. After dehydration, samples were infiltrated with a mixture of ethanol and acrylic resin in decreasing ratio (alcohol/resin ratio of 3:1, 1:1, 1:3, pure resin) and embedded in pure methyl methacrylate resin (Technovit 7200 VLC, Exact Kulzer, Bio-Optica, Milano, Italy).

### 2.2. Histomorphometric Analysis

The embedded samples were cut with a mounted diamond blade, ground to achieve a thickness of 70 microns, and stained with toluidine blue on a hot plate. Each sample was analyzed under a light microscope (Eclipse E600, Nikon, Tokyo, Japan) equipped with a calibrated digital camera (DXM1200, Nikon) at different magnifications (4×, 10×, 20×, and 40×), and digitally recorded for histomorphometric analysis (tissue fractions) of the regenerated area by ImageJ software (National Institutes of Health, Bethesda, MD, USA), similar to a previous report [20]. The proportions of lamellar bone, woven bone, osteoid, biomaterial particles, and medullary spaces were recorded for each patient.

Implants were loaded after 5 months of implant insertion.

### 2.3. Outcome Variables

The primary aim was to analyze a possible relationship between the primary outcome, i.e., histomorphometric fractions (evaluated as percentage of the total area examined), and the subantral bone height measured before the sinus surgery. A separate correlation analysis was performed for each graft material.

The residual bone height was determined in cross-sectional image sections from CBCT (J. Morita Corp., 3-33-18 Tarumi-cho, Suita-shi, Osaka 564-8650, Japan) acquired with a resolution of more than 2.5 LP/mm MTF. This initial (T1) CBCT examination was exported as a DICOM volume. The present study considered, as done in a previous study [14], only the grafted areas for the histomorphometric analysis, with the aim of being as representative as possible of the implant insertion site.

The secondary outcomes were implant survival, following the 2017 Periodontology World Workshop criteria [21], and marginal bone level change, evaluated 1 year after the prosthetic loading.

### 2.4. Statistical Analysis

Statistical analysis was performed in IBM SPSS Statistics 25 software. A *p*-value < 0.05 was considered significant. The Shapiro–Wilk test confirmed the normal distribution of the data. Descriptive statistics were calculated for each group, and Student’s t-test was used to compare preoperative RBH between groups. The Pearson correlation coefficient was calculated to assess the correlation between residual alveolar bone and each histomorphometric compartment proportion. Linear regression was used to evaluate the relationship of RBH with each histomorphometric result, separately for each group.

## 3. Results

The sample was composed of 20 subjects (12 males and 8 females), with a mean age of 59.1 ± 9.4 years. In each group, six males and four females were present. The mean age did not differ significantly between groups.

No significant differences in height of residual bone were found between the two groups (mean = 2.17 mm, minimum = 0.5 mm, maximum = 3.5 mm, SD = 1.11 mm in xenograft enriched with gelatin and polymer group; mean = 2.14 mm, minimum = 0.5 mm, maximum = 3.0 mm, SD = 0.72 mm in mineralized deproteinized bovine bone group, *p*-value = 0.4). RBH ranged from 0.5 to 3.5 mm.

**Figure 1:**
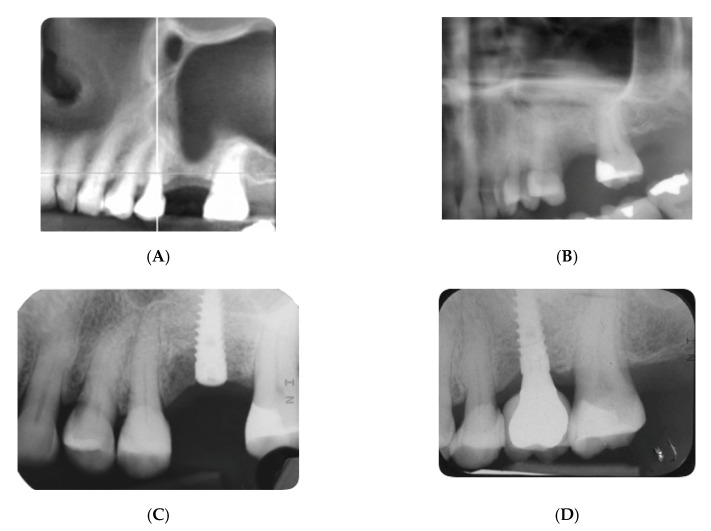
Radiographic images of a case with a residual bone height of 1 mm: (**A**) presurgical cone beam computed tomography (CBCT) showing the posterior residual bone; (**B**) image taken soon after the procedure of lateral sinus lift; (**C**) implant positioned after 5 months; (**D**) 1-year follow-up after loading.

**Figure 2:**
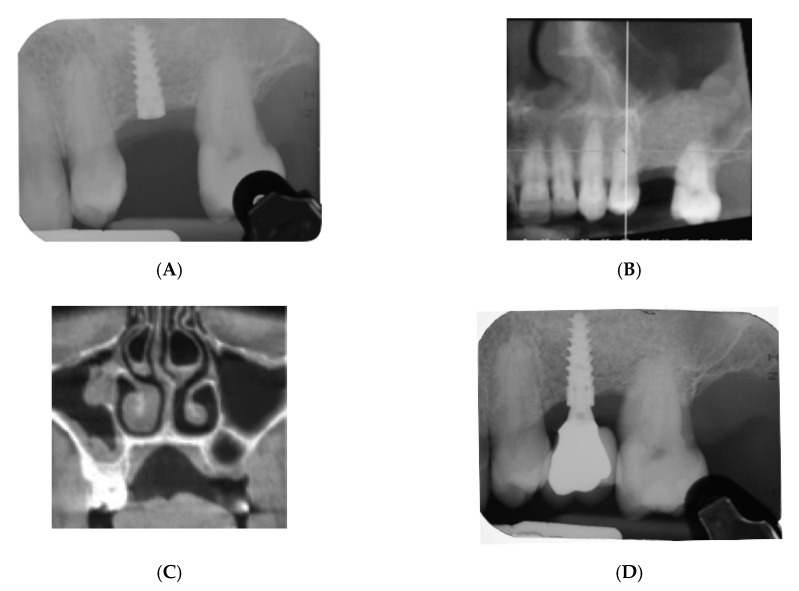
Radiographic images of a case with a residual bone height of 3.5 mm: (**A**) periapical radiograph showing posterior residual bone; (**B**) image taken soon after the procedure of lateral sinus lift; (**C**) implant positioned after 5 months; (**D**) 1-year follow-up after loading.

Figure 1 and Figure 2 show two cases with RBH of 1 and 3.5 mm, respectively.

**Figure 3:**
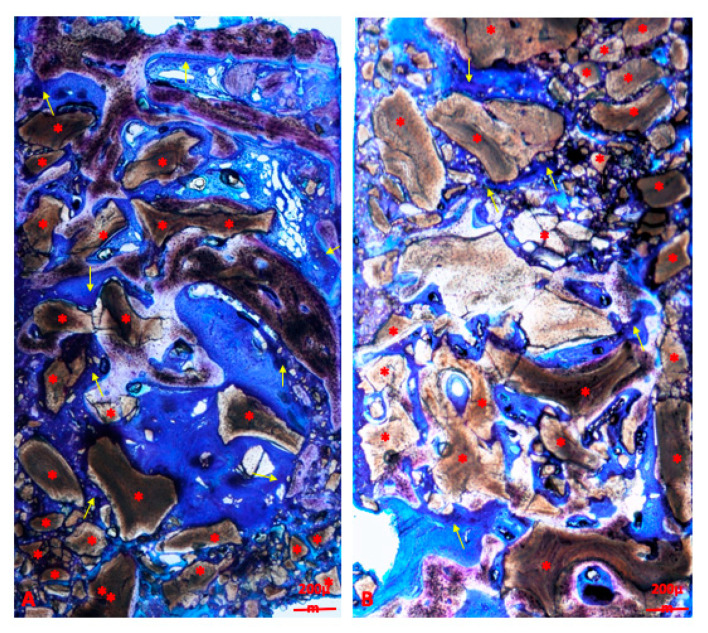
Overview of samples of the mineralized deproteinized bovine bone (DBMM) group (bar scale = 200 microns). Red asterisks are placed on blocks of biomaterials and yellow arrows indicate osteoid matrix. Blocks are surrounded by newly formed lamellar bone at different phases of mineralization. Blocks are easily recognizable and differentiable by bone that appears stained in shades of light brown and violet. (**A**,**B**) present differences in biomaterial amount, more represented in (**A**) than in (**B**), and consequent quantity of newly formed bone. Toluidine blue and pyronine yellow staining.

Detailed results of histological analysis were published by Taschieri et al. [14]. In brief, the grafted bone substitute appeared surrounded by newly formed bone and was slightly mineralized at 6 months. In the group grafted by a spongy mineralized deproteinized bovine bone (Bio-Oss, Geistlich Pharma AG, Wolhusen, Switzerland) histomorphometric analysis showed 23.14 ± 10.62% lamellar bone, 19.43 ± 9.18% woven bone, 23.35 ± 6.04% osteoid, 17.16 ± 6.13% biomaterial particles, and 16.93 ± 9.78% medullary spaces. An overview of a histological sample of the DBBM group is shown in Figure 3.

**Figure 4:**
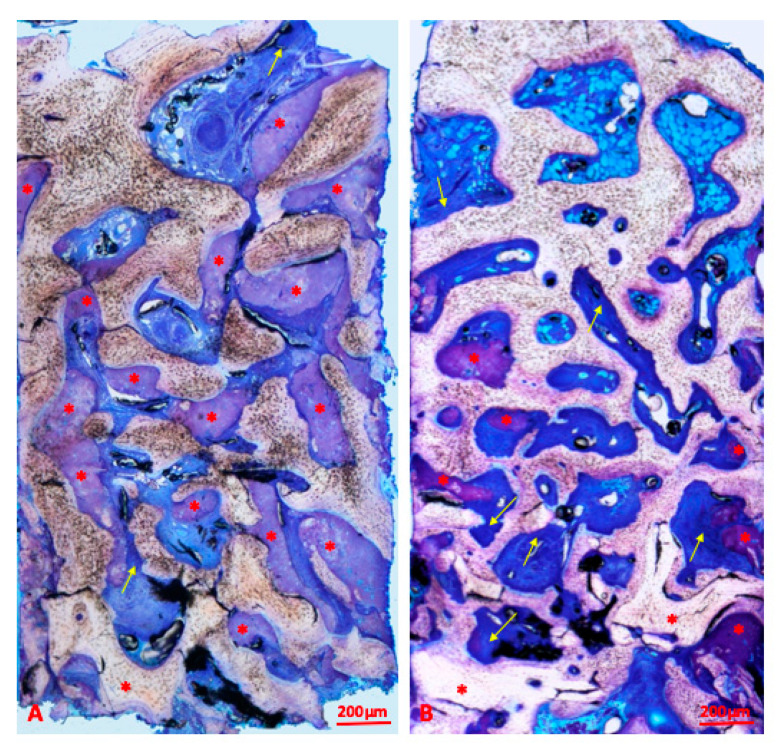
Overview of samples of the xenograft enriched by polymer and gelatin (NBS) group (bar scale = 200 microns). Red asterisks are placed on polymers and blocks of biomaterials while yellow arrows indicate osteoid matrix on the borders of the bony trabeculae. The residual biomaterial is stained in violet and is in close contact with newly formed lamellar bone at different phases of mineralization. New bone is organized in thick trabeculae interconnected among them. (**A**,**B**) present differences in biomaterial amount; biomaterial is more represented in (**A**) than in (**B**). Toluidine blue and pyronine yellow staining.

In the group grafted by an innovative hybrid xenograft enriched with gelatin and a polymer, the analysis showed 39.64 ± 12.02% lamellar bone, 16.28 ± 7.75% woven bone, 17.51 ± 4.87% osteoid, 12.72 ± 5.36% biomaterial particles, and 13.84 ± 6.53% medullary spaces. An overview of a histological sample of the NBS group is shown in Figure 4.

The study found significant differences between groups in proportion of lamellar bone (*p*-value = 0.004) and osteoid (*p*-value = 0.0287).

Results of the linear correlation between RBH and each fraction for each group are presented in Table 1.

In patients grafted with xenograft enriched with gelatin and polymer, RBH was observed to be positively correlated with woven bone and osteoid and negatively correlated with lamellar bone, biomaterial, and medullary spaces.

In patients grafted with mineralized deproteinized bovine bone, RBH was observed to be positively correlated with woven bone and lamellar bone and negatively correlated with osteoid, biomaterial, and medullary spaces.

The analysis showed the absence of a statistically significant difference in correlation between RBH and lamellar bone, woven bone, and osteoid in both groups. There were differences in the strength and direction of the correlation between the two groups.

Results of linear regression (Table 2) showed that 59% of the variability of woven bone, 50% of the variability of medullary spaces, and 31% of osteoid variability can be explained by RBH. Woven bone increased by 5.08% per 1-mm increment of RBH, medullary spaces decreased by 9.02% per 1-mm increase of RBH, and osteoid decreased by 4.4% per 1-mm increase of RBH in sinuses grafted by mineralized deproteinized bovine bone. In comparison, only 17% of lamellar bone variability can be explained by RBH, with lamellar bone increasing by 5.68% with each 1-mm increase of RBH.

Similar results were observed in sinuses grafted by xenograft enriched with gelatin and polymer: 45% of woven bone variability and 28% of lamellar bone variability can be explained by RBH. Woven bone increased by 8.08% per 1-mm increase of RBH, and lamellar bone decreased by 5.50% per 1-mm increase of RBH. On the other hand, only 10% of osteoid variability (osteoid increasing by 1.34% with each 1-mm of RBH) and less than 1% of the variability of medullary spaces (medullary spaces decreasing with each 1-mm increase of RBH) can be explained by RBH.

Implant success and survival rates were 100% 1 year after implant loading. The peri-implant bone loss averaged 1.02 ± 0.42 mm in the DBMM group and 0.95 ± 0.31 mm in the NBS group.

The results did not correlate to the age or the sex of the subjects, as evaluated statistically by regression analysis (*p* > 0.05).

## 4. Discussion

The current investigation used a prediction equation in order to explain histological results (lamellar bone, woven bone, osteoid biomaterial, and medullary spaces) of using different biomaterials, and the main findings are useful for clinical purposes in general. This method is applied starting from residual bone height (RBH), which is taken into account as a variable measured before maxillary sinus augmentation prior to dental rehabilitation.

In the literature, different techniques with or without the application of biologically active proteins, such as bone morphogenetic protein (BMP) or autologous platelet concentrates, are suggested for this procedure [22,23,24]. The proteins are in conjunction with the lateral antrostomy technique or the crestal approach (transcrestal sinus floor elevation), with simultaneous (single-stage surgery) or delayed (two-stage surgery) implant insertion [3,25]. Moreover, it has been recently proposed by several researchers to carry out the sinus floor elevation without grafting material, leaving the blood clot only to induce the natural healing processes [26,27,28,29]. Since the Tatum Jr. publication in 1986 [30], many studies have been performed on sinus lift outcomes, but to the authors’ knowledge, this is the first study concerning the application of a prediction equation to forecast histologic and histomorphometric outcomes of the sinus augmentation procedure by using the actual residual thickness of the subantral bone as a predictor. In the literature, only pooled values were reported, making this assessment difficult [26,28,29,31]. It is the authors’ conviction that RBH is one of the main factors able to influence a decision-making process and, therefore, the clinical outcomes of the sinus floor elevation procedure.

Some authors have found that with RBH lower or higher than 3 mm (taken as arbitrary threshold), the use of grafting material gave no significant advantage relative to the blood clot alone [28,32,33], but the outcomes were only clinical in all cases.

The biologic basis for the healing process is analogous to the classic postextraction socket healing. Inside this cavity, there is the formation of the fibrin mesh; the migration, proliferation, and differentiation of leukocytes, inflammatory cells, osteoclasts and osteoblasts, angiogenesis, and neovascularization of the blood clot; and the subsequent formation of woven bone and, consequently, new lamellar bone tissue. Engineered biomaterials can enhance this process by using their intrinsic osteoinductive properties to promote osteogenesis [26,29,33] and, therefore, influence the clinical decision-making process. Furthermore, this process allows the complete formation of new bone. Moreover, it reduces the clinical time that elapses until the rehabilitation of the patient with the dental prosthesis. The previously explained model can be used to compare and study all the innovative materials and techniques that could be developed in the near future.

Avila et al. in 2010 [19] assessed the influence of the distance from the lateral to the medial wall of the maxillary sinus on the outcomes of sinus augmentation procedures using a histomorphometric analysis. The authors observed that the percentage of vital bone formation after maxillary sinus augmentation was inversely proportional to the sinus buccopalatal distance. Histologic preparation and histomorphometric analysis were too different from the authors’ present study to be compared.

Similar results were presented by Stacchi et al. in 2018 in a histomorphometric study on 50 consecutive patients who were treated with transcrestal sinus floor elevation [34]. They found that the buccopalatal sinus width negatively affected the percentage of newly formed bone, suggesting that the latter can be strongly influenced by the sinus cavity dimensions [34]. However, the differences in the clinical protocol between transcrestal and lateral approaches for maxillary sinus augmentation make the study by Stacchi et al. hardly comparable to the present one.

In 2019, Pignaton et al. [35] investigated the influence of residual bone height and sinus width on the outcome of maxillary sinus bone augmentation using anorganic bovine bone. The conclusion of this study was similar to that of the present study; the posterior residual bone height and sinus width were not found to be influencing factors on new bone formation in sinuses grafted exclusively with anorganic bovine bone after 8 months of healing.

This study showed that there was a potential correlation between each 1-mm increment of RBH and the amount of newly formed woven bone, independent of the grafting material that was used. This correlation could lead the clinician to assume that the presence of RBH ranging between 1 and 2 mm could adversely affect the quality of graft regeneration and potentially increase the chances of failure of implant-supported prosthetic rehabilitation, especially when the implant is placed under function. Longer times may be required to allow for graft maturation and implant osseointegration in the case of extremely resorbed posterior maxilla.

The differences in lifestyle habits and anatomical and vascular features among patients make it very difficult to compare the data of the present report with those of similar previous studies.

Obviously, generally speaking, the sample size influences the *p*-value but has a minor effect on the statistical correlation. Moreover, the analysis of only one variable in the prediction model is the main limitation of this study, but the same statistical method might be applied to further specifically designed studies to gain more insight into the effect of other intrinsic variables on the outcome of maxillary sinus augmentation procedures.

## 5. Conclusions

The analysis showed the absence of a statistically significant difference in correlation of RBH and lamellar bone, woven bone, and osteoid independently of material used. In both groups, woven bone increased and medullary space decreased with each 1-mm increment of RBH. Osteoid and lamellar bone increased and decreased, respectively, with RBH increase in the NBS group, while the inverse was observed in the DBBM group.

## Figures and Tables

**Table 1:** Results of linear correlation between residual alveolar bone height (RBH) and each fraction for each group. Pearson correlation coefficients (*r*) are indicated.

	Mineralized Deproteinized Bovine Bone		Xenograft Enriched with Gelatin and Polymer	
	*r*	*p*-Value	*r*	*p*-Value
Lamellar bone	0.41	0.241	−0.53	0.113
Woven bone	0.67	0.034	0.77	0.009
Osteoid	−0.56	0.095	0.32	0.365
Biomaterial	−0.04	0.908	−0.13	0.728
Medullary spaces	−0.70	0.023	−0.07	0.853

**Table 2:** Results of linear regression.

	Mineralized Deproteinized Bovine Bone			Xenograft Enriched with Gelatin and Polymer		
	R Squared	Beta Coefficient	*p*-Value	R Squared	Beta Coefficient	*p*-Value
Lamellar bone	0.17	5.68	0.241	0.28	−5.50	0.113
Woven bone	0.59	5.08	0.034	0.45	8.08	0.009
Osteoid	0.31	−4.4	0.095	0.10	1.34	0.365
Biomaterial	0.002	−0.34	0.908	0.02	−0.58	0.728
Medullary spaces	0.50	−9.02	0.023	0.01	−0.38	0.853
